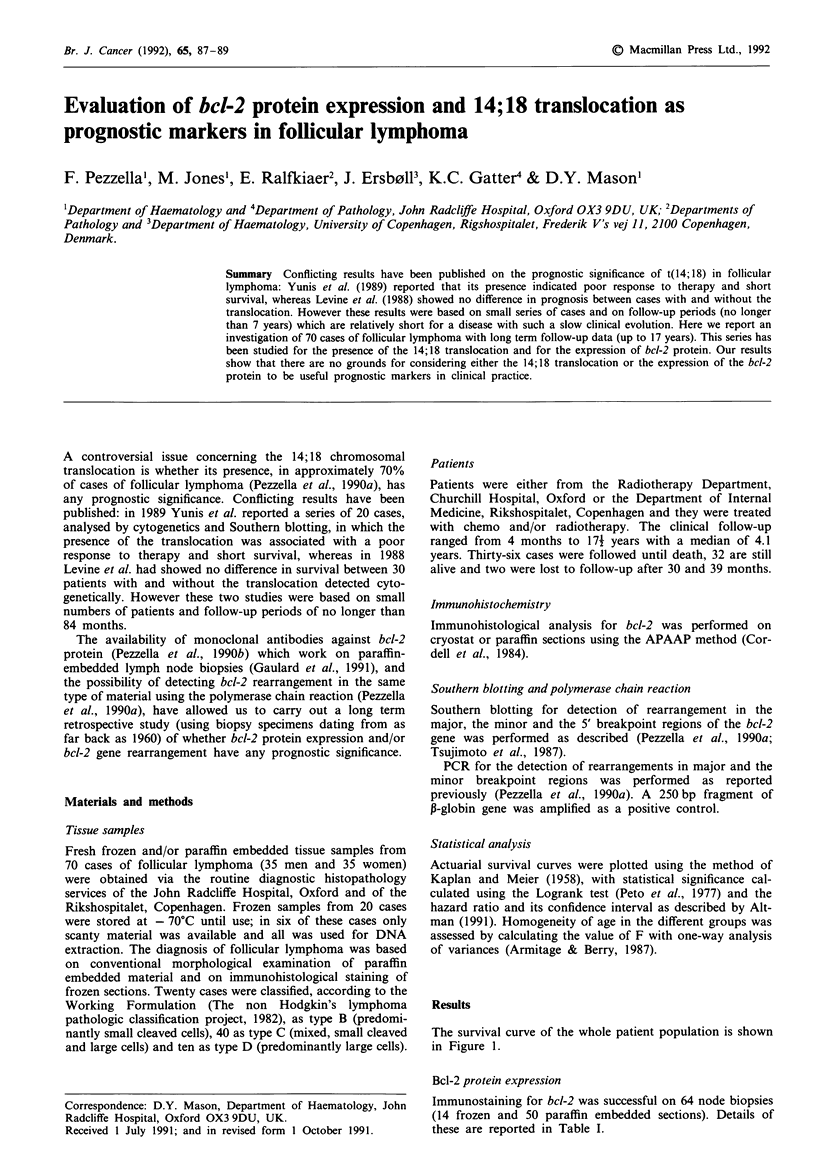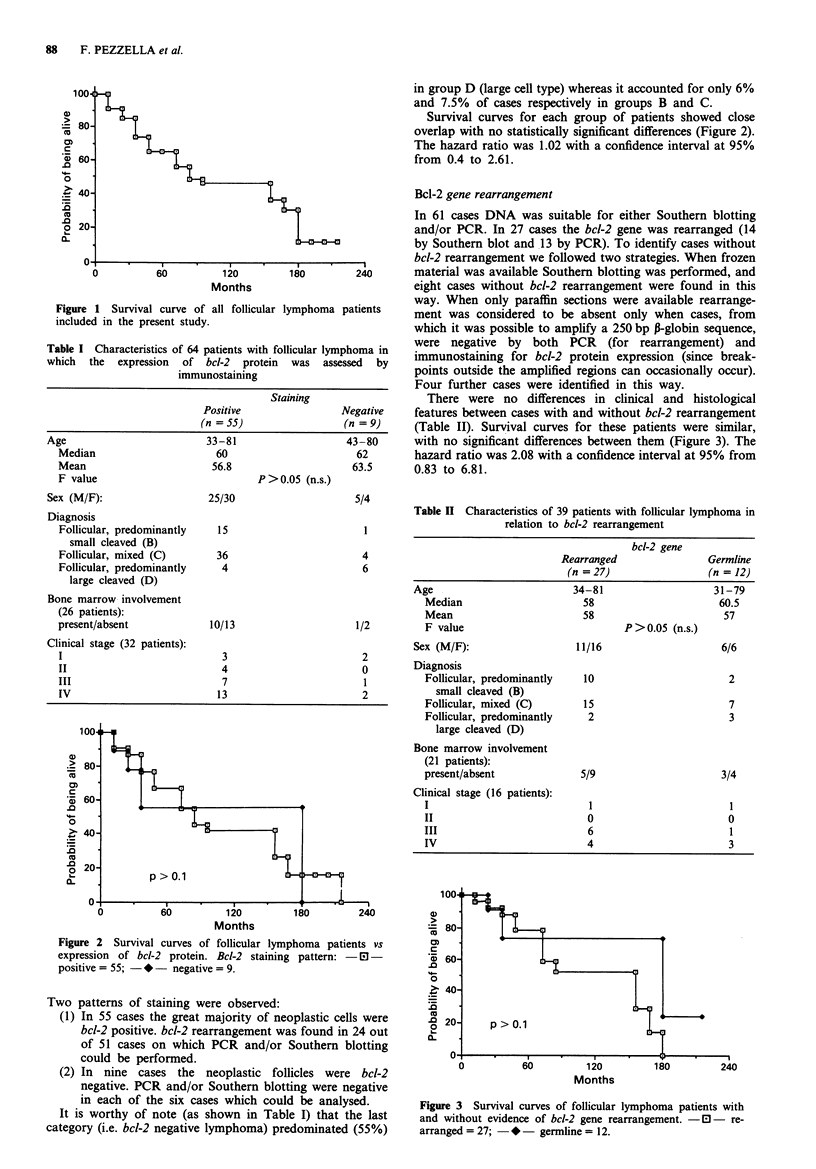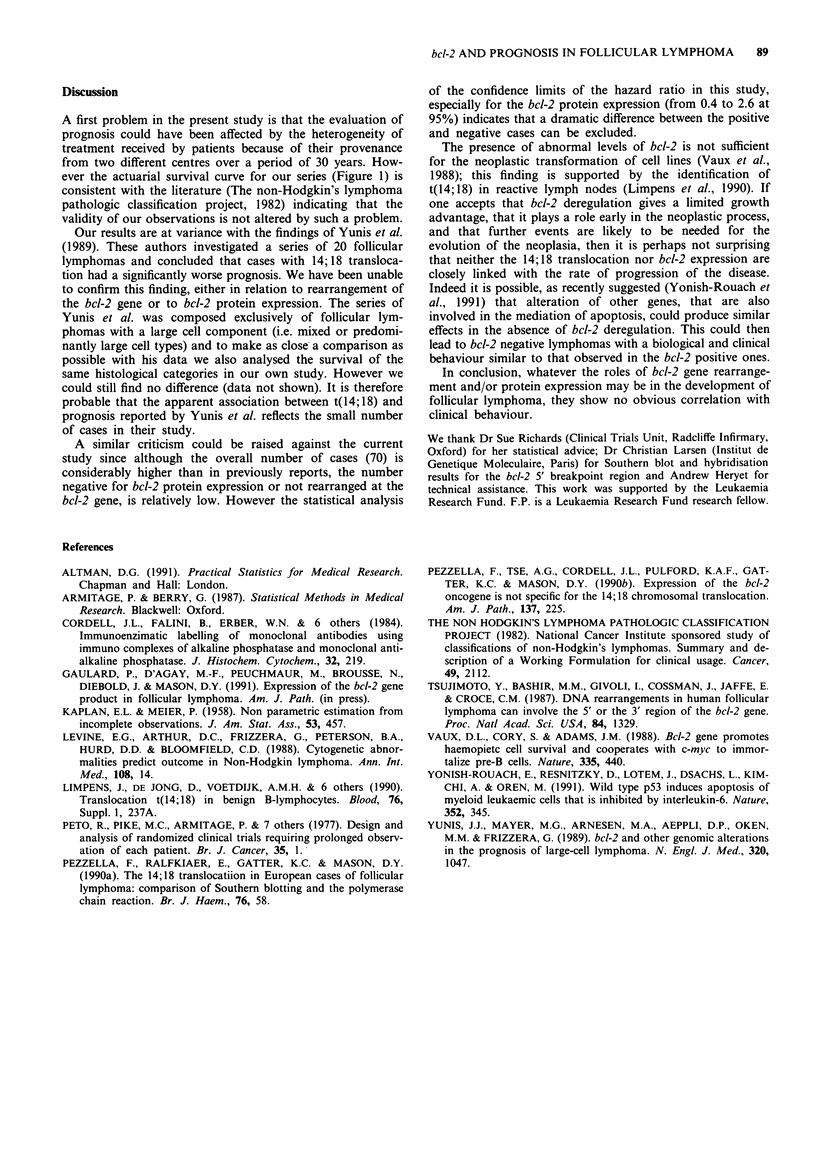# Evaluation of bcl-2 protein expression and 14;18 translocation as prognostic markers in follicular lymphoma.

**DOI:** 10.1038/bjc.1992.16

**Published:** 1992-01

**Authors:** F. Pezzella, M. Jones, E. Ralfkiaer, J. Ersbøll, K. C. Gatter, D. Y. Mason

**Affiliations:** Department of Haematology, John Radcliffe Hospital, Oxford, UK.

## Abstract

Conflicting results have been published on the prognostic significance of t(14;18) in follicular lymphoma: Yunis et al. (1989) reported that its presence indicated poor response to therapy and short survival, whereas Levine et al. (1988) showed no difference in prognosis between cases with and without the translocation. However these results were based on small series of cases and on follow-up periods (no longer than 7 years) which are relatively short for a disease with such a slow clinical evolution. Here we report an investigation of 70 cases of follicular lymphoma with long term follow-up data (up to 17 years). This series has been studied for the presence of the 14;18 translocation and for the expression of bcl-2 protein. Our results show that there are no grounds for considering either the 14;18 translocation or the expression of the bcl-2 protein to be useful prognostic markers in clinical practice.


Br. J. Cancer (1992), 65, 87–89 © Macmillan Press Ltd., 1992

### Evaluation of *bcl-2* protein expression and 14;18 translocation as prognostic markers in follicular lymphoma

F. Pezzella[1], M. Jones[1], E. Ralfkiaer[2], J. Ersbøll[3], K.C. Gatter[4] & D.Y. Mason[1]

[1]*Department of Haematology and* [4]*Department of Pathology, John Radcliffe Hospital, Oxford OX3 9DU, UK;* [2]*Departments of Pathology and* [3]*Department of Haematology, University of Copenhagen, Rigshospitalet, Frederik V's vej 11, 2100 Copenhagen, Denmark.*

**Summary** Conflicting results have been published on the prognostic significance of t(14;18) in follicular lymphoma: Yunis *et al.* (1989) reported that its presence indicated poor response to therapy and short survival, whereas Levine *et al.* (1988) showed no difference in prognosis between cases with and without the translocation. However these results were based on small series of cases and on follow-up periods (no longer than 7 years) which are relatively short for a disease with such a slow clinical evolution. Here we report an investigation of 70 cases of follicular lymphoma with long term follow-up data (up to 17 years). This series has been studied for the presence of the 14;18 translocation and for the expression of *bcl-2* protein. Our results show that there are no grounds for considering either the 14;18 translocation or the expression of the *bcl-2* protein to be useful prognostic markers in clinical practice.

A controversial issue concerning the 14;18 chromosomal translocation is whether its presence, in approximately 70% of cases of follicular lymphoma (Pezzella *et al.*, 1990*a*), has any prognostic significance. Conflicting results have been published: in 1989 Yunis *et al.* reported a series of 20 cases, analysed by cytogenetics and Southern blotting, in which the presence of the translocation was associated with a poor response to therapy and short survival, whereas in 1988 Levine *et al.* had showed no difference in survival between 30 patients with and without the translocation detected cytogenetically. However these two studies were based on small numbers of patients and follow-up periods of no longer than 84 months.

The availability of monoclonal antibodies against *bcl-2* protein (Pezzella *et al.*, 1990*b*) which work on paraffin-embedded lymph node biopsies (Gaulard *et al.*, 1991), and the possibility of detecting *bcl-2* rearrangement in the same type of material using the polymerase chain reaction (Pezzella *et al.*, 1990*a*), have allowed us to carry out a long term retrospective study (using biopsy specimens dating from as far back as 1960) of whether *bcl-2* protein expression and/or *bcl-2* gene rearrangement have any prognostic significance.

### Materials and methods

### *Tissue samples*

Fresh frozen and/or paraffin embedded tissue samples from 70 cases of follicular lymphoma (35 men and 35 women) were obtained via the routine diagnostic histopathology services of the John Radcliffe Hospital, Oxford and of the Rikshospitalet, Copenhagen. Frozen samples from 20 cases were stored at −70°C until use; in six of these cases only scanty material was available and all was used for DNA extraction. The diagnosis of follicular lymphoma was based on conventional morphological examination of paraffin embedded material and on immunohistological staining of frozen sections. Twenty cases were classified, according to the Working Formulation (The non Hodgkin's lymphoma pathologic classification project, 1982), as type B (predominantly small cleaved cells), 40 as type C (mixed, small cleaved and large cells) and ten as type D (predominantly large cells).

### *Patients*

Patients were either from the Radiotherapy Department, Churchill Hospital, Oxford or the Department of Internal Medicine, Rikshospitalet, Copenhagen and they were treated with chemo and/or radiotherapy. The clinical follow-up ranged from 4 months to 17½ years with a median of 4.1 years. Thirty-six cases were followed until death, 32 are still alive and two were lost to follow-up after 30 and 39 months.

### *Immunohistochemistry*

Immunohistological analysis for *bcl-2* was performed on cryostat or paraffin sections using the APAAP method (Cordell *et al.*, 1984).

### *Southern blotting and polymerase chain reaction*

Southern blotting for detection of rearrangement in the major, the minor and the 5′ breakpoint regions of the *bcl-2* gene was performed as described (Pezzella *et al.*, 1990*a*; Tsujimoto *et al.*, 1987).

PCR for the detection of rearrangements in major and the minor breakpoint regions was performed as reported previously (Pezzella *et al.*, 1990*a*). A 250 bp fragment of β-globin gene was amplified as a positive control.

### *Statistical analysis*

Actuarial survival curves were plotted using the method of Kaplan and Meier (1958), with statistical significance calculated using the Logrank test (Peto *et al.*, 1977) and the hazard ratio and its confidence interval as described by Altman (1991). Homogeneity of age in the different groups was assessed by calculating the value of F with one-way analysis of variances (Armitage & Berry, 1987).

### Results

The survival curve of the whole patient population is shown in Figure 1.

### Bcl-2 *protein expression*

Immunostaining for *bcl-2* was successful on 64 node biopsies (14 frozen and 50 paraffin embedded sections). Details of these are reported in Table I.

Correspondence: D.Y. Mason, Department of Haematology, John Radcliffe Hospital, Oxford OX3 9DU, UK.
Received 1 July 1991; and in revised form 1 October 1991.

**Figure 1** Survival curve of all follicular lymphoma patients included in the present study.

**Table I** Characteristics of 64 patients with follicular lymphoma in which the expression of *bcl-2* protein was assessed by immunostaining

| | *Staining* | | |
|---|---|---|---|
| | *Positive (n = 55)* | | *Negative (n = 9)* |
| Age | 33–81 | | 43–80 |
| Median | 60 | | 62 |
| Mean | 56.8 | | 63.5 |
| F value | | *P* > 0.05 (n.s.) | |
| Sex (M/F): | 25/30 | | 5/4 |
| Diagnosis | | | |
| Follicular, predominantly small cleaved (B) | 15 | | 1 |
| Follicular, mixed (C) | 36 | | 4 |
| Follicular, predominantly large cleaved (D) | 4 | | 6 |
| Bone marrow involvement (26 patients): present/absent | 10/13 | | 1/2 |
| Clinical stage (32 patients): | | | |
| I | 3 | | 2 |
| II | 4 | | 0 |
| III | 7 | | 1 |
| IV | 13 | | 2 |

**Figure 2** Survival curves of follicular lymphoma patients *vs* expression of *bcl-2* protein. *Bcl-2* staining pattern: —◻— positive = 55; —◆— negative = 9.

Two patterns of staining were observed:

(1) In 55 cases the great majority of neoplastic cells were *bcl-2* positive. *bcl-2* rearrangement was found in 24 out of 51 cases on which PCR and/or Southern blotting could be performed.

(2) In nine cases the neoplastic follicles were *bcl-2* negative. PCR and/or Southern blotting were negative in each of the six cases which could be analysed.

It is worthy of note (as shown in Table I) that the last category (i.e. *bcl-2* negative lymphoma) predominated (55%) in group D (large cell type) whereas it accounted for only 6% and 7.5% of cases respectively in groups B and C.

Survival curves for each group of patients showed close overlap with no statistically significant differences (Figure 2). The hazard ratio was 1.02 with a confidence interval at 95% from 0.4 to 2.61.

### Bcl-2 *gene rearrangement*

In 61 cases DNA was suitable for either Southern blotting and/or PCR. In 27 cases the *bcl-2* gene was rearranged (14 by Southern blot and 13 by PCR). To identify cases without *bcl-2* rearrangement we followed two strategies. When frozen material was available Southern blotting was performed, and eight cases without *bcl-2* rearrangement were found in this way. When only paraffin sections were available rearrangement was considered to be absent only when cases, from which it was possible to amplify a 250 bp β-globin sequence, were negative by both PCR (for rearrangement) and immunostaining for *bcl-2* protein expression (since breakpoints outside the amplified regions can occasionally occur). Four further cases were identified in this way.

There were no differences in clinical and histological features between cases with and without *bcl-2* rearrangement (Table II). Survival curves for these patients were similar, with no significant differences between them (Figure 3). The hazard ratio was 2.08 with a confidence interval at 95% from 0.83 to 6.81.

**Table II** Characteristics of 39 patients with follicular lymphoma in relation to *bcl-2* rearrangement

| | *bcl-2 gene* | | |
|---|---|---|---|
| | *Rearranged (n = 27)* | | *Germline (n = 12)* |
| Age | 34–81 | | 31–79 |
| Median | 58 | | 60.5 |
| Mean | 58 | | 57 |
| F value | | *P* > 0.05 (n.s.) | |
| Sex (M/F): | 11/16 | | 6/6 |
| Diagnosis | | | |
| Follicular, predominantly small cleaved (B) | 10 | | 2 |
| Follicular, mixed (C) | 15 | | 7 |
| Follicular, predominantly large cleaved (D) | 2 | | 3 |
| Bone marrow involvement (21 patients): present/absent | 5/9 | | 3/4 |
| Clinical stage (16 patients): | | | |
| I | 1 | | 1 |
| II | 0 | | 0 |
| III | 6 | | 1 |
| IV | 4 | | 3 |

**Figure 3** Survival curves of follicular lymphoma patients with and without evidence of *bcl-2* gene rearrangement. —◻— rearranged = 27; —◆— germline = 12.

### Discussion

A first problem in the present study is that the evaluation of prognosis could have been affected by the heterogeneity of treatment received by patients because of their provenance from two different centres over a period of 30 years. However the actuarial survival curve for our series (Figure 1) is consistent with the literature (The non-Hodgkin's lymphoma pathologic classification project, 1982) indicating that the validity of our observations is not altered by such a problem.

Our results are at variance with the findings of Yunis *et al.* (1989). These authors investigated a series of 20 follicular lymphomas and concluded that cases with 14;18 translocation had a significantly worse prognosis. We have been unable to confirm this finding, either in relation to rearrangement of the *bcl-2* gene or to *bcl-2* protein expression. The series of Yunis *et al.* was composed exclusively of follicular lymphomas with a large cell component (i.e. mixed or predominantly large cell types) and to make as close a comparison as possible with his data we also analysed the survival of the same histological categories in our own study. However we could still find no difference (data not shown). It is therefore probable that the apparent association between t(14;18) and prognosis reported by Yunis *et al.* reflects the small number of cases in their study.

A similar criticism could be raised against the current study since although the overall number of cases (70) is considerably higher than in previously reports, the number negative for *bcl-2* protein expression or not rearranged at the *bcl-2* gene, is relatively low. However the statistical analysis of the confidence limits of the hazard ratio in this study, especially for the *bcl-2* protein expression (from 0.4 to 2.6 at 95%) indicates that a dramatic difference between the positive and negative cases can be excluded.

The presence of abnormal levels of *bcl-2* is not sufficient for the neoplastic transformation of cell lines (Vaux *et al.*, 1988); this finding is supported by the identification of t(14;18) in reactive lymph nodes (Limpens *et al.*, 1990). If one accepts that *bcl-2* deregulation gives a limited growth advantage, that it plays a role early in the neoplastic process, and that further events are likely to be needed for the evolution of the neoplasia, then it is perhaps not surprising that neither the 14;18 translocation nor *bcl-2* expression are closely linked with the rate of progression of the disease. Indeed it is possible, as recently suggested (Yonish-Rouach *et al.*, 1991) that alteration of other genes, that are also involved in the mediation of apoptosis, could produce similar effects in the absence of *bcl-2* deregulation. This could then lead to *bcl-2* negative lymphomas with a biological and clinical behaviour similar to that observed in the *bcl-2* positive ones.

In conclusion, whatever the roles of *bcl-2* gene rearrangement and/or protein expression may be in the development of follicular lymphoma, they show no obvious correlation with clinical behaviour.

We thank Dr Sue Richards (Clinical Trials Unit, Radcliffe Infirmary, Oxford) for her statistical advice; Dr Christian Larsen (Institut de Genetique Moleculaire, Paris) for Southern blot and hybridisation results for the *bcl-2* 5′ breakpoint region and Andrew Heryet for technical assistance. This work was supported by the Leukaemia Research Fund. F.P. is a Leukaemia Research Fund research fellow.

### References

ALTMAN, D.G. (1991). *Practical Statistics for Medical Research*. Chapman and Hall: London.

ARMITAGE, P. & BERRY, G. (1987). *Statistical Methods in Medical Research*. Blackwell: Oxford.

CORDELL, J.L., FALINI, B., ERBER, W.N. & 6 others (1984). Immunoenzimatic labelling of monoclonal antibodies using immuno complexes of alkaline phosphatase and monoclonal anti-alkaline phosphatase. *J. Histochem. Cytochem.*, **32**, 219.

GAULARD, P., D'AGAY, M.-F., PEUCHMAUR, M., BROUSSE, N., DIEBOLD, J. & MASON, D.Y. (1991). Expression of the *bcl-2* gene product in follicular lymphoma. *Am. J. Path.* (in press).

KAPLAN, E.L. & MEIER, P. (1958). Non parametric estimation from incomplete observations. *J. Am. Stat. Ass.*, **53**, 457.

LEVINE, E.G., ARTHUR, D.C., FRIZZERA, G., PETERSON, B.A., HURD, D.D. & BLOOMFIELD, C.D. (1988). Cytogenetic abnormalities predict outcome in Non-Hodgkin lymphoma. *Ann. Int. Med.*, **108**, 14.

LIMPENS, J., DE JONG, D., VOETDIJK, A.M.H. & 6 others (1990). Translocation t(14;18) in benign B-lymphocytes. *Blood*, **76**, Suppl. 1, 237A.

PETO, R., PIKE, M.C., ARMITAGE, P. & 7 others (1977). Design and analysis of randomized clinical trials requiring prolonged observation of each patient. *Br. J. Cancer*, **35**, 1.

PEZZELLA, F., RALFKIAER, E., GATTER, K.C. & MASON, D.Y. (1990a). The 14;18 translocatiion in European cases of follicular lymphoma: comparison of Southern blotting and the polymerase chain reaction. *Br. J. Haem.*, **76**, 58.

PEZZELLA, F., TSE, A.G., CORDELL, J.L., PULFORD, K.A.F., GATTER, K.C. & MASON, D.Y. (1990*b*). Expression of the *bcl-2* oncogene is not specific for the 14;18 chromosomal translocation. *Am. J. Path.*, **137**, 225.

THE NON HODGKIN'S LYMPHOMA PATHOLOGIC CLASSIFICATION PROJECT (1982). National Cancer Institute sponsored study of classifications of non-Hodgkin's lymphomas. Summary and description of a Working Formulation for clinical usage. *Cancer*, **49**, 2112.

TSUJIMOTO, Y., BASHIR, M.M., GIVOLI, I., COSSMAN, J., JAFFE, E. & CROCE, C.M. (1987). DNA rearrangements in human follicular lymphoma can involve the 5′ or the 3′ region of the *bcl-2* gene. *Proc. Natl Acad. Sci. USA*, **84**, 1329.

VAUX, D.L., CORY, S. & ADAMS, J.M. (1988). *Bcl-2* gene promotes haemopietc cell survival and cooperates with c-*myc* to immortalize pre-B cells. *Nature*, **335**, 440.

YONISH-ROUACH, E., RESNITZKY, D., LOTEM, J., DSACHS, L., KIMCHI, A. & OREN, M. (1991). Wild type p53 induces apoptosis of myeloid leukaemic cells that is inhibited by interleukin-6. *Nature*, **352**, 345.

YUNIS, J.J., MAYER, M.G., ARNESEN, M.A., AEPPLI, D.P., OKEN, M.M. & FRIZZERA, G. (1989). *bcl-2* and other genomic alterations in the prognosis of large-cell lymphoma. *N. Engl. J. Med.*, **320**, 1047.